# Optimization of Transcranial Direct Current Stimulation of Dorsolateral Prefrontal Cortex for Tinnitus: A Non-Linear Dose-Response Effect

**DOI:** 10.1038/s41598-018-26665-1

**Published:** 2018-05-29

**Authors:** Giriraj Singh Shekhawat, Sven Vanneste

**Affiliations:** 1 Section of Audiology and Health Systems, University of Auckland, Auckland, New Zealand; 2 Center for Brain Research, University of Auckland, Auckland, New Zealand; 3 Tinnitus Research Initiative, Regensburg, Germany; 4 Lab for Clinical & Integrative Neuroscience, School of Behavioral and Brain Sciences, University of Texas, Dallas, USA; 5 Center for Brain Health, University of Texas at Dallas, Richardson, TX USA; 6 Callier Center of Communication Disorders, University of Texas at Dallas, Richardson, TX USA

## Abstract

Neuromodulation is defined as the process of augmenting neuroplasticity via invasive or non-invasive methods. Tinnitus is the perception of sound in the absence of its external source. The objective of this study was to optimize the parameters of transcranial direct current stimulation (tDCS) of dorsolateral prefrontal cortex (DLPFC) for tinnitus suppression. The following factors were optimized in the dose-response design (*n* = 111): current intensity (1.5 mA or 2 mA), stimulation duration (20 min or 30 min), and number of stimulation sessions (2, 4, 6, 8, or 10), with a 3–4 day washout period between each session. Participants underwent a minimum of 2 sessions in 1 week or maximum of 10 sessions in 5 weeks’ time. Tinnitus loudness was measured in pre-post design using a 10-point numeric rating scale. There was a significant reduction in tinnitus loudness after tDCS of DLPFC. There was no significant difference between the intensity and duration of stimulation. As the number of sessions increased, there was a higher reduction in the tinnitus loudness; however, this effect plateaued after 6 sessions.

## Introduction

Neuromodulation is defined as the process of augmenting neuroplasticity by using invasive or non-invasive methods^1^. Several non-invasive neuromodulation techniques—such as transcranial magnetic stimulation (TMS), neurofeedback, transcranial direct current stimulation (tDCS), and transcutaneous electrical nerve stimulation (TENS)—have been used as tools for researching various health conditions^2^. Research on using neuromodulation to treat these conditions is in its infancy, but early studies have shown great promise. The primary goal of neuromodulation is to augment neuroplasticity, so that the brain can be primed to respond better to management. It is hypothesized to work via modulating neuronal excitability and/or synaptic strength, disturbing the neural networks underlying the pathology^1,3^. The primary focus of the present study is tDCS. TDCS has been used widely for various neuropsychiatric conditions such as anxiety, depressive disorders, schizophrenia, obsessive-compulsive disorder, chronic pain, and Parkinson’s disease^4^. However, we still lack a complete understanding of the mechanism underlying tDCS^5^ and how to optimize it for specific conditions.

TDCS has been applied to more than 3,000 individuals without any significant adverse effects using the following protocol: current intensity = 1–2 mA; electrode size = 25–35 cm^2^; and stimulation duration = 20–30 minutes per session^6–9^. TDCS is a well-tolerated and comfortable technique. The most common side effects are mild tingling and light itching sensations under the electrodes, during the stimulation^10,11^. Depending upon the polarity of the stimulation, tDCS can increase or decrease cortical excitability in the stimulated brain region. Anodal stimulation elicits an excitatory effect due to neuronal depolarization; cathodal stimulation conversely elicits and inhibitory effect due to neuronal hyperpolarization^12^.

Initial studies have demonstrated the duration and strength of the tDCS after-effects are dependent upon the intensity and duration of stimulation^13^. Nearly linear effects of stimulation intensity (0.2–2.0 mA) and duration (1–13 min) were found^14^. Since these early studies, a tendency in tDCS research is to increase duration and intensity of stimulation with the anticipation of greater outcome effects^15^. However, the number of studies looking at the duration and intensity are limited. Furthermore, most studies do not account for the number of sessions needed to induce a therapeutic effect in patients. Each of these parameters is potentially relevant for clinical outcomes.

Take, for instance, the application of tDCS to tinnitus. Tinnitus is an auditory phantom sensation experienced when no external sound is present^16^, and is commonly described as ringing, buzzing, cricket-like, hissing, whistling, and/or humming^17^. Tinnitus is a highly prevalent condition^18^ and adversely affects the overall quality of life of sufferers^19^. Tinnitus perception is the result of change in neuronal activity^20,21^. The first published evidence of tinnitus suppression by tDCS came in 2006^22^. Since then, there have been several studies confirming the transient suppressive effect of tDCS on tinnitus^23^. TDCS has been used as a research tool for transient tinnitus suppression for at least a decade^23^. However, to convert tDCS from a research tool to a clinical tool, more research is needed in the direction of converting the transient impact into a lasting one^23^. Garin *et al*.^24^ were the first to document lasting after-effects of tDCS, but these long-term effects could not be replicated. Garin suggested a need for optimization of tDCS parameters for long-term tinnitus relief.

**Figure 1:**
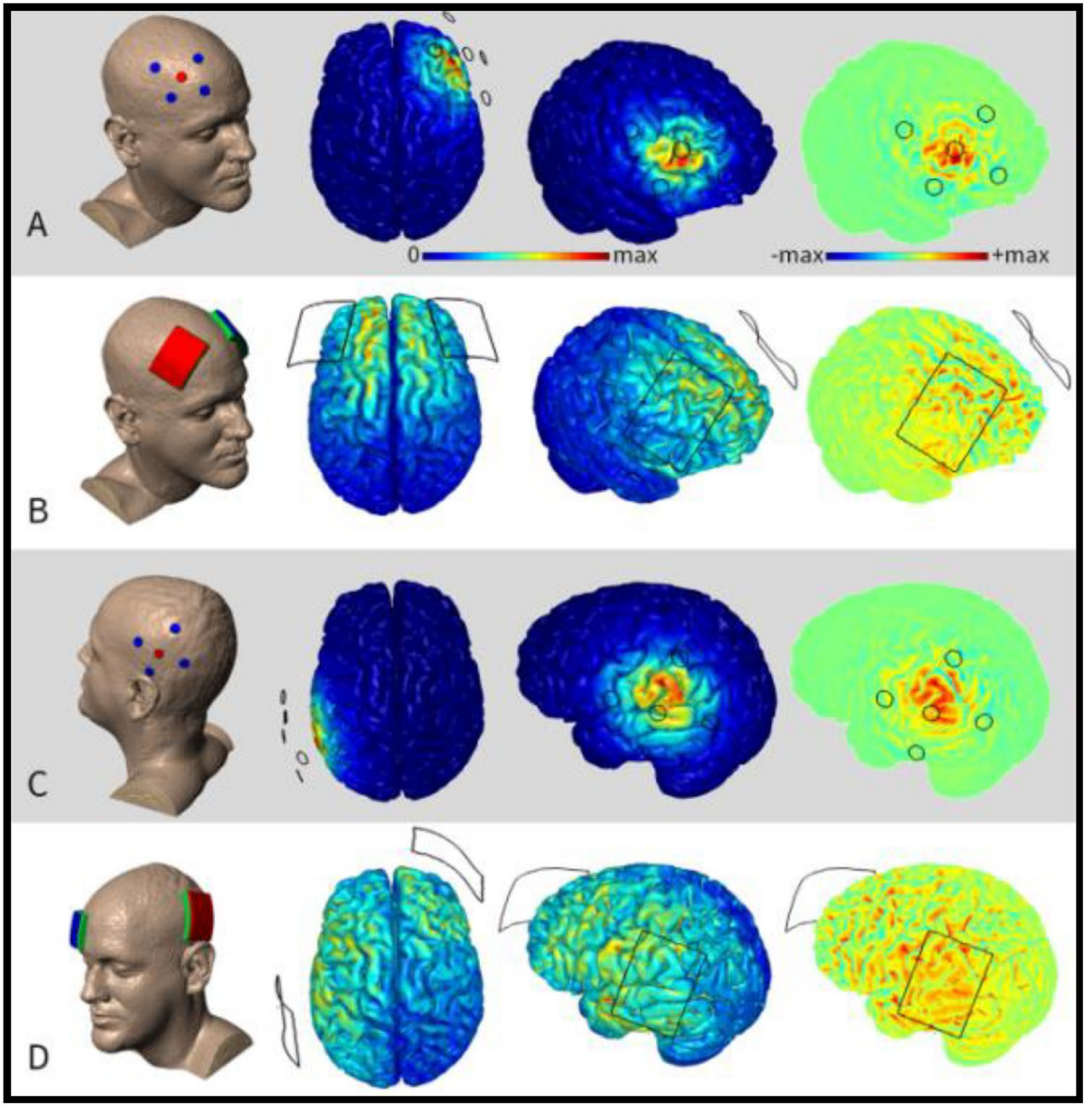
Finite element models of transcranial direct current stimulation montages nominally targeting DLPFC (**A**,**B**) and LTA (**C**,**D**). Taken from Shekhawat *et al*.^25^.

Optimizing the settings of tDCS is necessary to maximize the impact of tDCS on tinnitus suppression. There has been some early research in the area of optimizing tDCS stimulation parameters (intensity, duration, and location) for tinnitus suppression^25–27^. Shekhawat *et al*.^26^, for example, conducted a dose-response study and found that tDCS of left temporoparietal area (LTA) with a 2-mA current intensity and a 20-minute duration is the most effective setting for transient tinnitus suppression. In another study, Shekhawat *et al*.^25^ found both LTA and dorsolateral prefrontal cortex (DLPFC) to be effective stimulation sites for transient tinnitus suppression. This study also conducted a human head modeling experiment demonstrating the distribution of electric field during non-invasive brain stimulation as shown in Fig. 1. Furthermore, there are some early trends^23^ supporting the stimulation of LTA for tinnitus loudness suppression and DLPFC stimulation for tinnitus annoyance suppression. DLPFC not only contains auditory memory cells but has also been associated with auditory attention, early inhibitory modulation of input to primary auditory cortex, and a facilitatory effect on auditory memory storage^28–32^. DLPFC is actively involved in modulating tinnitus loudness and annoyance^9,33^. The optimization trial by Shekhawat *et al*.^26^ looked specifically at LTA as the site of stimulation; there is no evidence about DLPFC optimization as the site of stimulation. We therefore decided to stimulate DLPFC for the present study.

There is no literature addressing the frequency of tDCS sessions needed for optimal tinnitus suppression. Thus, there are several open questions: how many sessions of tDCS are needed for optimal results? What should the washout period be between the tDCS sessions? What would be the outcome of head-to-head comparisons of multiple sessions of tDCS? The current trial was therefore designed with the aim to optimize tDCS of DLPFC by investigating the impact of current intensity (1.5 mA or 2 mA), duration (20 minutes or 30 minutes), and number of sessions (2, 4, 6, 8, or 10) on tinnitus suppression.

## Material and Methods

### Participants

Participants were recruited through the Tinnitus Research Initiative (TRI), Antwerp, Belgium. Data were collected in the context of a clinical treatment and are retrospectively analyzed here. Participants provided written informed consent in accordance with Declaration of Helsinki that their data could be used for research purposes. The study was approved by the ethical committee of University of Antwerp, Belgium and was carried out in accordance with the approved guidelines. Participants were eligible for inclusion if they were 18 years or older with chronic tinnitus for a minimum of 6 months. Participants were excluded if they had any contraindications to undergo tDCS such as: metal implants in the head or body, epilepsy, heart condition, pregnancy, psychotropic medications as screened by a neurologist.

One hundred eleven participants (70 male; mean age = 61.14 years ± 13.78 and 41 female mean age = 56.88 years ± 14.60) with average tinnitus duration of 5.9 years completed this trial. Demographic details of participants included in this study are documented in Table 1. Forty-seven participants had bilateral tinnitus, 28 localized it in left ear, 18 perceived it in the right ear, and 18 felt it was in the head. For 26 participants, the quality of their tinnitus was pure tone and 85 participants felt their tinnitus to be like narrow band noise. See Table 1 for information.

**Table 1:** Demographic details of the study participants.

Age	Gender	Tinnitus		
		Duration	Type	Localization
59.87	70 Male	5.9 Years	26 Pure tone	47 Bilateral
Years	41		85 Narrow band	18 Head
	Female		noise	28 Left ear
				18 Right ear

### Procedure

This was a dose-response study aiming to optimize the number of tDCS sessions needed to suppress tinnitus perception. Participants underwent tDCS of DLPFC with two current intensities (1.5 mA or 2 mA) and two durations (20 minutes or 30 minutes) based on the trial conducted by Shekhawat *et al*.^26^. There were five variables in the ‘sessions’ used (2, 4, 6, 8, or 10 sessions). These variables were selected based on the scoping review conducted by Shekhawat *et al*.^23^. Participants underwent a minimum of 2 sessions over the course of a week and maximum of 10 sessions over 5 weeks’ time. The participants were randomly assigned. Based on the registration of participants for this trial, they were randomly allocated to undergo one of the five stimulation session options (e.g., the first person undergoes 2 sessions; the second, 6 sessions; the third, 10 sessions; the fourth, 4 sessions; the fifth, 8 sessions; and so on).

### Clinical Evaluation

Tinnitus loudness was evaluated using a 10-point numeric rating scale, where 1 to 10 represented a spectrum of tinnitus from ‘very quiet’ (1) to ‘as loud as imaginable’ (10) based on the scoping review conducted by Shekhawat *et al*.^23^. Tinnitus loudness was assessed before and after the end tDCS sessions.

### Transcranial Direct Current Stimulation

TDCS was performed using a specially developed, battery-driven, constant current stimulator with a maximum output of 10 mA (Eldith; http://www.eldith.de). Rubber electrodes with a surface area of 35 cm^2^ were encased in electrode sponges soaked in NaCl solution (0.85%) based on Dundas *et al*.^34^. The cathode was placed over the left DLPFC (F3) and the anode over the right DLPFC (F4) as determined by the international 10/20 Electroencephalogram System. Sham stimulation was not used in this study as the focus was to optimize the number of sessions required to suppress tinnitus perception in a dose-response manner.

### Data Analysis

Statistical analysis was performed using the SPSS 22.0 software package. A repeated measures ANOVA with within-subjects (pre- vs. post-stimulation tinnitus loudness rating scale) and between-subjects sessions (2, 4, 6, 8, and 10 sessions), current intensity (1.5 or 2 mA), and stimulation duration (20 or 30 minutes). We additionally conducted a univariate ANOVA with the percentage of change on the tinnitus loudness rating scale as the dependent variable and sessions, intensity, and duration as independent variables. Furthermore, an independent t-test was used to compare the effect of the number of sessions on the percentage of change on the tinnitus loudness rating scale. We conducted another independent t-test to compare the effect of stimulation intensity and stimulation duration for the 6 sessions only. At the request of one reviewer, we also added a generalized linear model including sessions, current intensity, and stimulation duration as independent variables and percent change on the tinnitus loudness rating scale as dependent variable.

We applied a one-way ANOVA with sessions, current intensity, and stimulation duration as independent variables and responder rate (10% drop on the loudness rating scale as well as 1 point drop on the loudness rating scale) as the dependent variable. We further ran an independent t-test to compare the effect of stimulation duration for responder rate for each number of sessions separately. We also added a generalized linear model with sessions, current intensity, and stimulation duration as independent variables and responder rate (10% drop on the numeric rating scale as well as 1 point drop on the numeric rating scale) as the dependent variable.

## Results

### Suppression effect

**Figure 2:**
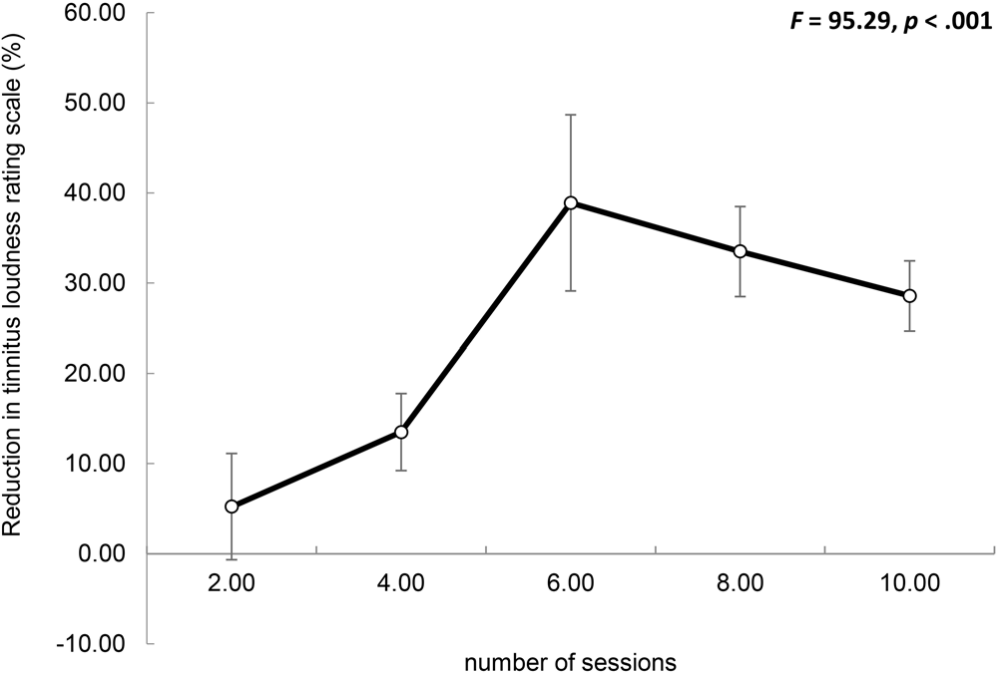
Reduction in tinnitus loudness rating scale over the number of tDCS sessions. Error bar represents ± 1 SD change in the mean. Higher values represent more reduction in tinnitus loudness and a negative value represents worsening in tinnitus loudness.

A repeated-measures ANOVA yielded a significant effect for pre- vs. post-stimulation tinnitus loudness numeric rating scale (*F*(1,93) = 95.29, *p* < 0.001) showing a significant reduction in tinnitus loudness after tDCS treatment (*M* = 6.48, *Sd* = 1.98) in comparison to before treatment (*M* = 4.91, *Sd* = 2.17). This effect was moderated by the number of sessions (*F*(4,93) = 8.36, *p* < 0.005). However, no interaction effect was obtained for either the intensity of stimulation (*F*(1,93) = 0.14, *p* = 0.71) or the duration (*F*(1,93) = 0.19, *p* < 0.66). Figure 2 shows the reduction in tinnitus loudness rating scale over the different number of tDCS sessions used. The maximum reduction was observed after 6 sessions of tDCS. For the percent change, a significant effect was obtained for the number of sessions (*F*(4,93) = 5.59, *p* = 0.02) but not for either the intensity (*F*(1,93) = 0.62, *p* = 0.43) or the duration (*F*(1,93) = 1.81, *p* = 0.18). Table 2 shows the results of the independent t-test. There was a significant difference between 2 vs. 8 sessions (*t* = 3.69, *p* = 0.001) and between 4 vs. 8 sessions (*t* = 2.89, *p* = 0.006). No significant effect was obtained between 6 vs. 8 (*t* = 0.54, *p* = 0.59) or 8 vs. 10 sessions (*M* = 28.57, *Sd* = 21.70; *t* = 0.79, *p* = 0.43). A comparison between 2 vs. 4 sessions (*t* = 1.09, *p* = 0.28) revealed no significant effects. However, comparisons between 2 vs. 6 (*t* = 3.13, *p* = 0.004) and between 2 vs. 10 (*t* = 3.44, *p* = 0.001) sessions each revealed significant effects. In addition, comparisons between 4 vs. 6 sessions (*t* = 2.52, *p* = 0.017) and between 4 vs. 10 sessions (*t* = 2.48, *p* = 0.017) yielded significant differences. However, these two effects do not survive Bonferroni correction for multiple comparisons. No significant difference was obtained between 6 vs. 10 sessions (*t* = 1.18, *p* = 0.25).

**Table 2:** Result of independent t test showing difference between the sessions of tDCS.

	Sessions					Total
	2	4	6	8	10	
Percentage of Change SD	5.21^a^ (27.67)	13.49^a^ (18.15)	38.89^b^ (37.92)	33.49^b^ (24.93)	28.57^b^ (21.70)	24.00 (28.10)

Difference subscript indicates a significant difference.

**Figure 3:**
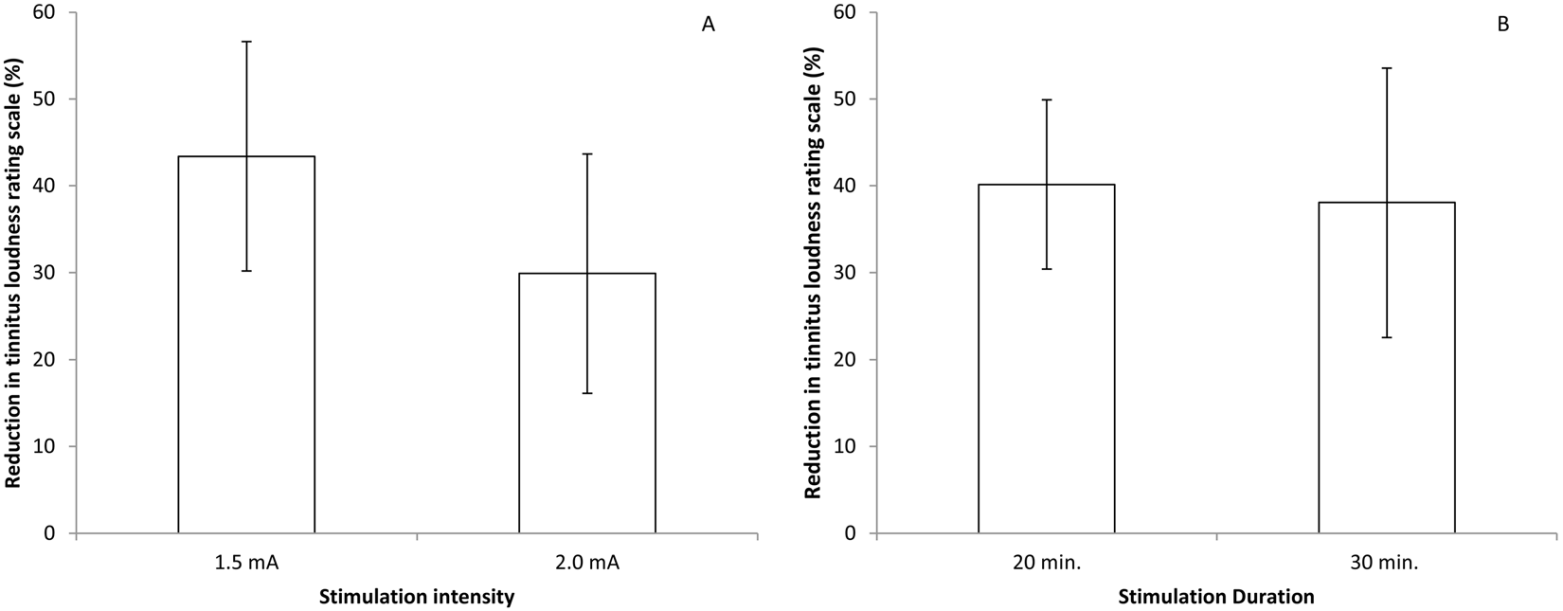
(**A**) For 6 sessions, a comparison between the stimulation intensity (1.5 and 2.0 mA) showed no significant difference; (**B**) For 6 sessions, a comparison between the stimulation duration (20 or 30 min) showed no significant difference.

We ran a repeated-measures ANOVA controlling for the tinnitus characteristics, namely side, type, gender, and age. This latter analysis did not show an effect for tinnitus characteristics. Similar to our previous analysis, we observed that 6 sessions creates the largest amount of suppression with the minimal amount of sessions. We further looked to see if there was a difference for stimulation intensity (1.5 or 2 mA) and stimulation duration (20 or 30 min.) for 6 sessions; however, our analysis revealed no significant effects for intensity (*t* = 0.64; *p* = 0.54) or duration (*t* = 0.10; *p* = 0.92) (see Fig. 3).

To verify the effect of the number of sessions on tinnitus suppression, we also applied a generalized linear model including sessions, current intensity, and stimulation duration as independent variables and percent change on the tinnitus loudness rating scale as the dependent variable. This analysis revealed an overall effect (omnibus test likelihood ratio χ^2^(6) = 16.63, *p* = 0.011) showing that the number of sessions has an effect (Wald χ^2^(4) = 11.91, *p* = 0.018) on the percent change on the tinnitus loudness rating scale. However, intensity (Wald χ^2^(1) = 0.014, *p* = 0.91) and duration (Wald χ^2^(1) = 0.08, *p* = 0.77) do not generate an effect. A closer look at the parameter estimates shows that both 2 (Wald χ^2^(1) = 6.06, *p* = 0.014) and 4 (Wald χ^2^(1) = 4.96, *p* = 0.026) sessions significantly differ from the other sessions. 6 (Wald χ^2^(1) = 0.31, *p* = 0.86) and 8 (Wald χ^2^(1) = 0.24, *p* = 0.63) sessions also differ from 10 sessions.

### Responder rate

**Figure 4:**
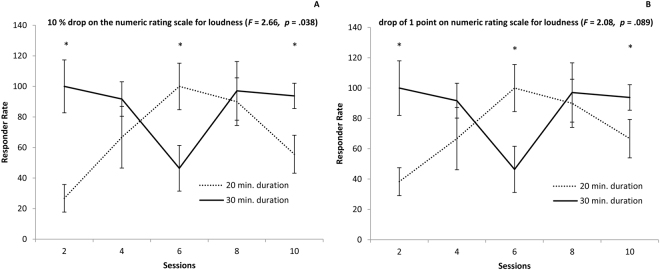
A comparison in the responder rate (**A**) 10% drop on the numeric rating scale for loudness; (**B**) drop of 1 point on the numeric rating scale for loudness) for the amount of sessions (from to 2 up to 10 sessions) × duration (20 vs. 30 minutes).

A one-way ANOVA with session, duration, and intensity as independent variables and responder rate (10% drop on the numeric rating scale) as the dependent variable revealed an effect for interaction between sessions x duration (*F*(4,93) = 2.66, *p* = 0.038). Specifically, our results demonstrate that more people respond to 6 sessions of 20 minutes than to 6 sessions of 30 minutes (*t* = 2.55, *p* = 0.024) (see Fig. 4). The opposite effect was shown for 2 (*t* = 2.07, *p* = 0.044) and 10 (*t* = 3.05, *p* = 0.005) sessions, but these do not survive correction for multiple corrections. No interaction was obtained for 4 and 8 sessions. No significant effects were obtained for the main effect of sessions, intensity, and duration or for the interaction effects of session × intensity, intensity × duration, and sessions × intensity × duration.

To verify the effect of sessions on tinnitus suppression we also applied a generalized linear model including sessions, current intensity, and stimulation duration as independent variables and responder rate (10% drop on the numeric rating scale) as the dependent variable. This revealed a significant effect (likelihood ratio χ^2^(9) = 41.12, *p* < 0.001) of omnibus test. We also found an interaction between sessions × duration (Wald χ^2^(9) = 24.61, *p* = 0.003). A pairwise comparison shows a significant difference between 6 sessions of 20 minutes and 6 sessions of 30 minutes (*p* = 0.036).

A similar analysis was conducted for responder rate (one-point drop) on the numeric rating scale demonstrating a marginally significant effect for sessions x duration (*F*(4,93) = 2.08, *p* = 0.089), indicating a trend that more people respond to 6 sessions of 20 minutes than to 6 sessions of 30 minutes (*t* = 2.55, *p* = 0.024) (see Fig. 3). The opposite was shown for 2 (*t* = 2.07, *p* = 0.044) and 10 (*t* = 2.28, *p* = 0.03) sessions, while no duration-related differences were obtained for 4 or for 8 sessions. The effect for 2 and 8 sessions do not remain after correction of multiple corrections. No effect was obtained for the main effect of sessions, intensity, and duration or for the interaction effects of session × intensity, intensity × duration, and sessions × intensity × duration.

To verify the effect of sessions on we also apply a generalized linear model including sessions, current intensity, and stimulation duration as independent variables and responder rate (one-point drop) as the dependent variable. This revealed a significant effect likelihood ratio (χ^2^(9) = 49.45, *p* < 0.001) of omnibus test. We find an interaction between sessions x duration (Wald χ^2^(9) = 28.17, *p* = 0.001) A pairwise comparison shows a significant differences between 6 sessions of 20 minutes and to 6 sessions of 30 minutes (*p* = 0.036).

## Discussion

Overall, there was a significant reduction in the tinnitus loudness after tDCS of DLPFC relative to before the treatment. Reduction in tinnitus loudness increased with the number of sessions, plateauing after 6 sessions. There was no significant difference between the intensities (1.5 mA or 2 mA) and durations (20 min and 30 min) of stimulation that we tested. Based on this study, the optimized setting for repeated tDCS of DLPFC to reduce tinnitus loudness would be: intensity = 1.5 mA; duration = 20 minutes; sessions = 6 sessions over 3 weeks’ time, with a washout period of 3–4 days between each session.

Few attempts have been made to study the impact of multiple sessions of tDCS on tinnitus suppression^35–39^. Most of these studies did not report any positive impact. Note, however, that these studies all used a similar protocol of five tDCS sessions on five consecutive days. It is possible that using five sessions on five consecutive days without longer intermittent periods cancels out the tinnitus suppressive effect observed after a single session of tDCS. Other studies suggest that applying repeated sessions produces cumulative changes in therapeutic applications^40^. Our data are consistent with this latter idea, showing that repeated stimulation results in cumulative changes in cerebral function, but that this effect was not linear. Six sessions clearly achieved a greater effect than 2 or 4 sessions but results did not improve with additional sessions. These findings will inform future clinical trials especially for tinnitus management where multiple stimulation sessions will be used.

Previous research already conducted a dose-response study to optimize the current intensity (1, 1.5, and 2 mA) and duration (10 and 20 min.) for tinnitus suppression^26^. However, that study only focused on LTA as the site of stimulation and did not attempt to optimize the number of sessions. TDCS of LTA with a current intensity of 2 mA and 20 minutes duration was the most effective setting in that study for transient tinnitus suppression. However, in the present study we explored DLPFC as the site of stimulation and specifically the optimum number of sessions for tinnitus suppression. The results of the present study recommend 20 minutes duration as optimum for DLPFC stimulation, with an intensity of 1.5 mA. This fits with previous research that did not show significant changes in higher vs. lower intensities resulting in identical excitability after-effects and short-latency intracortical inhibition^41^. This is also supported Bastani and Jaberzadeh, who reported a uniform effect of 0.3 and 2.0 mA on cortical excitability^42^. Initial studies showed an effect of tDCS dependent upon duration (from 1 to 13 min)^3,14^. Our study shows no difference in the amount of reduction for 20 vs. 30 minutes of stimulation, suggesting a non-linear saturation point where 30 minutes does not generate a greater effect than 20 minutes. Looking at the responder rate, 30 minutes of stimulation actually seems to induce a lower responding rate than 20 minutes. This fits with previous findings that suggest that stimulation duration of anodal tDCS increased the percentage of responders^13^. It is however not clear why 20 minutes works better than 30 minutes. However, previous trials looking at the optimization of neuromodulation were focused on HD-tDCS and compared both LTA and DLPFC as the site of stimulation^25^. The winning setting for tinnitus suppression in that trial was: 2 mA current intensity, 20 min duration, and both locations were effective in modulating tinnitus loudness and annoyance. A possible reason for this could be explained by the difference in research design and number of extra sessions participants underwent in the present trial (2–10 sessions), which was limited to only 2 sessions in the HD-tDCS trial.

Hyvärinen *et al*.^43^ conducted a double blind, sham controlled trial investigating the self-administered domiciliary tDCS treatment for tinnitus. Forty three participants with chronic tinnitus underwent tDCS of LTA and DLPFC for 10 sessions on 10 consecutive days. TDCS was not shown to be effective in tinnitus suppression, however. The washout period in this trial was <1 day, similar to other trials^35–39^ which did not find a positive impact of tDCS on tinnitus. This is further evidence that back-to-back sessions on consecutive days is not effective for tinnitus suppression and masks the impact of individual sessions. Contrary to the above studies, there were three trials that resulted in significant transient reduction in tinnitus^9,44^ and long term suppression of tinnitus^24^ using different washout periods. Faber *et al*.^9^ and Frank *et al*.^44^ conducted tDCS on alternative days (6 sessions in 2 weeks) after each session of anodal, cathodal and sham tDCS; Garin *et al*.^24^ used a two-week washout period. In the present study, we used a washout period of 3–4 days between each session of tDCS. We recommend >1 day washout period between tDCS sessions to sustain the impact on tinnitus suppression. Conducting the sessions on consecutive days does not facilitate the plastic changes that lead to tinnitus suppression.

### Future implications

The design of this trial was a dose-response paradigm, aiming to optimize multiple sessions of tDCS. We therefore did not use sham controlled sessions. For future research, we recommend double blind, sham controlled trials^45^ with the winning settings of the present study. It would also be interesting to incorporate a positive control such as a DLPFC-related task as a confirmation of target engagement. Multi-session tDCS (6 sessions) with a 3–4 day washout period exploring different sites of stimulation would also be valuable.

Finally, we recommend investigating the long-term impact of multiple session tDCS on tinnitus loudness and annoyance. It would be valuable to explore multi-session tDCS (6 sessions) by alternating the stimulation between LTA and DLPFC (odd sessions with LTA stimulation, even sessions with DLPFC stimulation). This could facilitate the stimulation of neuroanatomical structures between the LTA and DLPFC, resulting in stronger priming of the brain for tinnitus modulation. This could also be an effective method to overcome the technical limitations of simultaneously stimulating LTA and DLPFC.

## Conclusion

This study was the first attempt to optimize the settings of tDCS for DLPFC stimulation to modulate tinnitus, especially to optimize the number of sessions needed for tinnitus suppression. TDCS of DLPFC resulted in a significant reduction of tinnitus loudness. The optimal setting for repeated tDCS stimulation of DLPFC to reduce tinnitus loudness, as reported by our study, would be: intensity = 1.5 mA; duration = 20 minutes; sessions = 6 sessions over 3 weeks’ time with a washout period of 3–4 days. The present study revealed a non-linear cumulative change in cerebral function as a result of multisession tDCS. We recommend further research using randomized control design, investigating multiple stimulation of two different sites (LTA and DLPFC), and exploring the long term impact of tDCS on tinnitus loudness and annoyance. Furthermore, more biochemical, molecular, and behavioral evidence is needed to support our study’s conclusions.
